# Vaping, Environmental Toxicants Exposure, and Lung Cancer Risk

**DOI:** 10.3390/cancers15184525

**Published:** 2023-09-12

**Authors:** Shaimaa A. Shehata, Eman A. Toraih, Ezzat A. Ismail, Abeer M. Hagras, Ekramy Elmorsy, Manal S. Fawzy

**Affiliations:** 1 Department of Forensic Medicine and Clinical Toxicology, Faculty of Medicine, Suez Canal University, Ismailia 41522, Egypt; shaimaa_shehata@med.suez.edu.eg (S.A.S.); abeerhagras@med.suez.edu.eg (A.M.H.); 2 Division of Endocrine and Oncologic Surgery, Department of Surgery, School of Medicine, Tulane University, New Orleans, LA 70112, USA; etoraih@tulane.edu; 3 Genetics Unit, Department of Histology and Cell Biology, Faculty of Medicine, Suez Canal University, Ismailia 41522, Egypt; 4 Department of Urology, Faculty of Medicine, Suez Canal University, Ismailia 41522, Egypt; boezzat@med.suez.edu.eg; 5 Department of Pathology, Faculty of Medicine, Northern Border University, Arar 73213, Saudi Arabia; ekramy.elmorsy@nbu.edu.sa; 6 Department of Forensic Medicine and Clinical Toxicology, Faculty of Medicine, Mansoura University, Mansoura 35516, Egypt; 7 Department of Biochemistry, Faculty of Medicine, Northern Border University, Arar 73213, Saudi Arabia

**Keywords:** pulmonary, cancer, e-smoking, risk, environment, vaping, electronic cigarette

## Abstract

**Simple Summary:**

Lung cancer (LC) is considered one of the most common cancers globally. Numerous studies have determined the relations between E-cigarette, or vaping, products (EVPs) and many proven environmental toxicants in LC development. Even though tobacco smoke remains the chief cause of LC, there is increasing concern that EVPs use could also increase LC risk. Consumption of EVPs has been dramatically increasing world-wide, particularly among younger people and non-smokers. This review seeks to consolidate the known environmental toxicants and EVPs contributing to LC to ensure that future research endeavors may identify key focus areas. Thus, EVPs are a highly potential risk factor for LC and an area of significant concern for the future. Since these factors have been linked to the development of LC, more research is needed to determine the mechanisms by which they affect lung pathology. Discovering the pathophysiology of EVPs use and environmental toxicant exposure in LC development can facilitate the adoption of exposure reduction strategies.

**Abstract:**

Lung cancer (LC) is the second-most prevalent tumor worldwide. According to the most recent GLOBOCAN data, over 2.2 million LC cases were reported in 2020, with an estimated new death incident of 1,796,144 lung cancer cases. Genetic, lifestyle, and environmental exposure play an important role as risk factors for LC. E-cigarette, or vaping, products (EVPs) use has been dramatically increasing world-wide. There is growing concern that EVPs consumption may increase the risk of LC because EVPs contain several proven carcinogenic compounds. However, the relationship between EVPs and LC is not well established. E-cigarette contains nicotine derivatives (e.g., nitrosnornicotine, nitrosamine ketone), heavy metals (including organometal compounds), polycyclic aromatic hydrocarbons, and flavorings (aldehydes and complex organics). Several environmental toxicants have been proven to contribute to LC. Proven and plausible environmental carcinogens could be physical (ionizing and non-ionizing radiation), chemicals (such as asbestos, formaldehyde, and dioxins), and heavy metals (such as cobalt, arsenic, cadmium, chromium, and nickel). Air pollution, especially particulate matter (PM) emitted from vehicles and industrial exhausts, is linked with LC. Although extensive environmental exposure prevention policies and smoking reduction strategies have been adopted globally, the dangers remain. Combined, both EVPs and toxic environmental exposures may demonstrate significant synergistic oncogenicity. This review aims to analyze the current publications on the importance of the relationship between EVPs consumption and environmental toxicants in the pathogenesis of LC.

## 1. Introduction

Lung cancer (LC) is the second-most prevalent tumor worldwide and is considered the underlying cause of cancer-related death [1,2]. According to the most recent GLOBOCAN data, in 2020, there were 2,206,771 new cases of LC and 1,796,144 recorded deaths globally [3]. Lung cancer is the most prevalent cancer in males, followed by prostate and colorectal cancer [4]. While breast cancer is the most frequently diagnosed cancer in females, followed by LC and colorectal cancer [4]. Lung tumor is a global problem and is more frequent in 37 countries, including China, Russia, the Middle East, Eastern Europe, and Southeast Asia [5]. Despite significant advances in diagnostic strategies and effective new treatment lines, the 5-year survival rate of LC is only 10–20% [6]. Lung cancer has a poor prognosis, and more than 75% of lung cancers are diagnosed in a late advanced stage with multiple systemic metastatic, particularly in developing countries [7,8].

Several extrinsic and intrinsic factors play a significant role in LC pathogenesis. Extrinsic factors included lifestyle, environmental toxicants, occupational exposure, and some specific infections, while intrinsic influences involved sex, immune, and genetic factors [2,9,10,11]. The potential role of genetic sustainability and gene mutations and alteration in developing and progression of LC was described in numerous published studies [12,13,14]. It is proven that causally associate LC with active and passive smoking and various occupational and environmental toxic agents [10]. The respiratory tract is considered a sensitive organ that is characterized by a sizeable absorbent area exposed to several toxic agents. Exposure to these agents over time may eventually lead to oncogenesis in that tissue [15].

**Figure 1:**
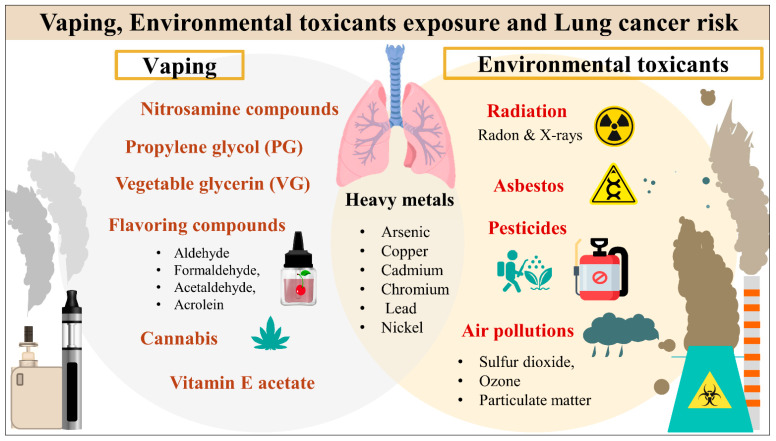
Vaping products and environmental toxicants exposure and lung cancer risk.

Traditional and electronic smoking, as well as further risk factors such as environmental toxicants, exposure to arsenic, asbestos, and air pollution, remain significant contributors to LC development as demonstrated in Figure 1 [5]. A more profound comprehension of the epidemiology and risk factors for LC can guide preventative strategies and reduce the rising disease burden globally [5,16]. E-cigarette, or vaping, products (EVPs) use has been dramatically increasing worldwide, remarkably among younger non-smokers and more females [17,18]. Unfortunately, adults aged 18 to 24 had the highest rate of EVP use, with over 2 million middle and high school students reporting use [19,20]. In the United States (US), a 900% surge in the use of EVPs among high school students was documented between 2011 and 2015 [20]. According to the Centers for Disease Control and Prevention (CDC) recent surveillance, electronic cigarettes (ECs) were the most widely used tobacco product among US teenagers in 2020 [21]. In 2019, CDC reported that over 2,500 patients were diagnosed with ECs, or vaping, product use-associated lung injury (EVALI), and they were males under 35 using vaping tetrahydrocannabinol (THC)-containing counterfeit street ECs products [22]. There is growing concern that EVPs consumption may increase the risk of LC because EVPs contain several proven carcinogenic compounds [15]. However, the relationship between EVPs and LC is not well established, and the long-term effects will take years to develop [15,23].

A recent study detected more than 500 chemicals in tested vaping cartridges, and most were categorized as carcinogens [24]. Basically, E-Liquid is composed of four main ingredients: nicotine, water, flavorings and humectants, propylene glycol (PG) and vegetable glycerin (VG) [25]. An ECs contains nicotine derivatives (e.g., nitrosnornicotine, nitrosamine ketone), heavy metals, and flavorings (aldehydes and complex organics) [24]. Other proved toxins such as formaldehyde, acrolein, acetaldehyde, metallic nanoparticles, benzene, toluene, ethylbenzene, and xylene [24,26,27]. The oncogenicity of EVPs has been attributed to several distinct molecular pathways. The direct chemical reactions or carcinogenic products generated by combustion and pyrolysis could induce oxidative stress, epithelial-mesenchymal transition, and mitochondrial DNA genotoxicity [23].

There is an increase in the incidence of LC among nonsmokers, which could be attributed to environmental exposure to several known toxic or carcinogenic compounds [10]. Because of global industrialization and increased pollution, the etiological factors of LC have become increasingly complex [10]. Chronic low-dose environmental toxicants exposures have been associated with various cancers, including thyroid carcinoma [28,29], liver [28], breast [30], bladder [31], skin [32], and kidney [33]. Several experimental studies revealed that various compounds found in the environment could induce cancer by triggering cellular, gene mutation and molecular alterations [34,35]. Numerous toxic agents were classified as carcinogenic to humans based on the Environmental Protection Agency of the United States (EPA) and the International Agency for Research on Cancer (IARC) [35,36,37]. Environmental health risks are related to chemical, physical, and biological factors. Among these compounds include pesticides, asbestos, particulate matter (PM), polychlorinated biphenyls (PCBs), polycyclic aromatic hydrocarbons, heavy metals and physical (ionizing and non-ionizing radiations such as exposure to radon or ultraviolet (UV) radiation, respectively) [10,38]. Also, prolonged environmental exposure to air pollution increases LC risk [39]. A study in the USA observed a 40% increased risk of LC among six US cities with the highest PM levels in the air [5]. According to the IACR, arsenic has been implicated in LC, and a 3.6-fold increased risk of LC mortality has been reported among individuals living in Chile who were exposed to increased arsenic levels in their drinking water during the 1950s–1970s [40]. Hence, arsenic and PM is classified as group I carcinogen by the IARC [37].

Despite various studies on the risk factors, classification, and pathogenesis of LC, little data is devoted to the interplay between EVPs consumption and environmental toxicants that determine the development of malignancy. Combined, both toxic exposures may demonstrate significant synergistic oncogenicity [41]. The present review aims to explore the current publications on the importance of the relationship between EVPs consumption and environmental toxicants in the pathogenesis of LC.

## 2. Materials and Methods

The association between environmental toxicants exposure, vaping, and lung cancer was investigated by looking for research in international databases such as Scopus, PubMed, and Web of Science. Vaping device/e-liquid contents, potential carcinogens, effects of vaping smoke/e-liquid products, environmental risk factors/exposure, lung, cancer, pesticides, persistent organic pollutants (POPs), including heavy metals, polychlorinated biphenyls (PCBs), bisphenol A (BPA), phthalates, and radiation were some of the keywords applied during the search. 

## 3. Epidemiology of Lung Cancer

Lung cancer is the second-most frequent malignance diagnosed worldwide and is considered one of the underlying causes of death [1,2]. According to the most recent GLOBOCAN data, over 2.2 million LC cases were reported in 2020, with an estimated new death incident of 1,796,144 lung cancer cases [3]. Sex variation, geographical differences, environmental contamination, occupational exposure, and histologic subtypes of LC also exhibited noticeable differences in incidence patterns [42]. Although LC is the most common male cancer, there are increasing trends in women’s LC incidence and mortality [43]. From its geographical distribution, Polynesia had the highest LC incidence, followed by Micronesia and Eastern Asia. While regarding the mortality rates, Micronesia had the highest mortality followed by Polynesia and Eastern Asia [43]. Early detection and recent advancements in LC targeting therapy decrease mortality in some high-income nations, including the United Kingdom, the United States, and Australia [43,44]. Several epidemiological studies and reports have stated that LC etiology is related to proven and potential risk factors [42]. Lung cancer risk has been connected to several jobs and industries, including metallurgy, driving, mining, and construction [45]. Smoking is the most significant and well-documented risk factor for LC. Smoking has been linked to more than 90% of LC in men, while occupational exposures are responsible for 10% to 20% [46,47]. A synergistic effect has been detected between several occupational exposures and smoking [45]. Occupational LC represents nearly 75% of all occupational cancers [47]. Smoking has been identified as the primary risk factor for LC in several studies [48,49,50]. Tobacco smoking is a known risk factor for lung cancer, with more than 70 human carcinogens identified based on the IARC report. Moreover, IARC monographs summarized the epidemiologic research findings supporting a causal link between tobacco use and lung cancer [51]. It has been observed that there is a stronger association between smoking and the LC types SCC and SCLC than adenocarcinoma and LCC [39]. It has been proven that exposure to secondhand tobacco smoke from parents or in the workplace is linked with an elevated risk of LC. However, the evidence linking childhood exposure to tobacco smoke and an increased risk of LC is scarce [52,53,54]. The mechanisms through which smoking and environmental exposures lead to raised risk of LC are not yet well established. It is suggested that exposure to carcinogenic agents could lead to oxidative stress, DNA damage, chronic inflammatory activity, growth factors, elevated cytokines, and DNA repair dysfunction [55].

**Figure 2:**
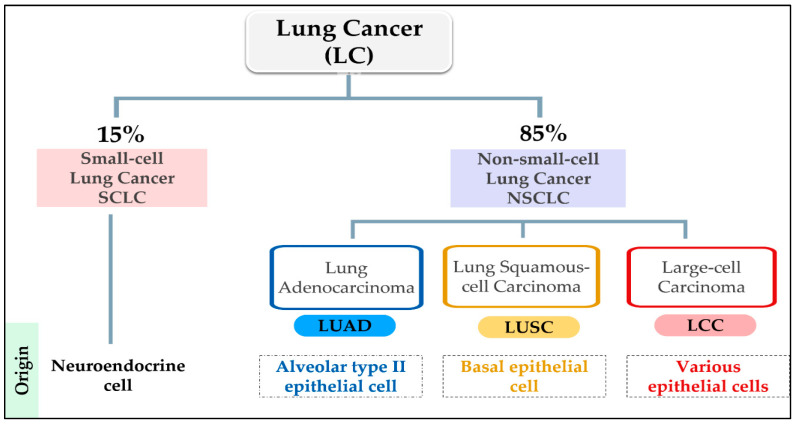
Histological classification of lung cancer (LC); Small-cell Lung Cancer (SCLC); Non-small cell Lung Cancer (NSCLC); Adenocarcinoma (LUAD); Squamous-cell Carcinoma (LUSC) and Large-cell Carcinoma (LCC).

Lung cancer is a heterogeneous illness with various clinicopathological characteristics [56]. Histologically, LC is categorized into two groups: small-cell lung (SCLC) and non-small-cell lung (NSCLC) [57]. The total diagnosis percentage for NSCLC is 85%, while only 15% for SCLC. The tumor origin of SCLC is poorly differentiated neuroendocrine, while NSCLC sub-types of cancer derived from lung epithelia. In addition, NSCLC is subdivided into three sub-types based mainly on the morphology of the transformed cells: adenocarcinoma (LUAD), squamous-cell carcinoma (LUSC), and large-cell carcinoma (LCC) [58]. The NSCLC subtypes developed from alveolar type II epithelial cells in LUAD and airway basal epithelial cells in LUSC [59] (Figure 2). According to the 2015 WHO classification, the most frequent subtype of LC is LUAD, followed by LUSC [60]. Unfortunately, the prognosis for all LC types is poor, specifically, LUSC and SCLC, which are predictably detected in tobacco-using male smokers [61]. The SCLC is characterized by rapid metastasis, poor prognosis, and poorly responsive to therapy [5,62].

Regarding genetic susceptibility to LC, it has been recognized that nearly 85% of LC risk was linked to cigarette smoking. Hence, LC develops in 15% of smokers, proposing a differential susceptibility to the effects of tobacco carcinogens. Additionally, 10% to 15% of LC arise in non-smokers. Differences in genetic profiles probably have a role in this differential sustainability [12]. In literature, synergized smoking and environmental toxicant exposures produced carcinogenic effects and were accompanied by several somatic mutations in LC. Known mutations and loss of heterozygosity in oncogenes and tumor suppressor genes involved in lung carcinogenesis accumulate in individual somatic cells during lung tumor initiation and progression [12,13,63]. The genetic complexity of LC was stated in numerous published articles. According to the Cancer Genome Atlas, sequencing 178 squamous cell carcinomas confirmed the complexity of LC, with a mean of 165 genomic rearrangements, 360 exonic mutations, and 323 copy number alterations per tumor [64]. In smokers, numerous mutations were identified, including Kirsten rat sarcoma (*KRAS*), tumor protein p53 (*TP53*), epidermal growth factor receptor (EGFR), v-raf murine sarcoma viral oncogene homolog B (*BRAF*), and serine/ threonine kinase 11 (*STK11*) [12]. Adenocarcinoma is more likely to arise in women and individuals with a smoking history. It occurs peripherally and tests positive for targetable driver mutations such as anaplastic lymphoma kinase (*ALK*), *EGFR*, *ROS1*, and *BRAF* [5,62]. LC continues to be the most prevalent underlying cause of cancer-related death. Although there has been rapid advancement in LC screening and target treatment, further research focusing on the genetic contribution to LC susceptibility is required.

## 4. Vaping and Environmental Toxicants Interact as Lung Cancer Risk Factors

**Figure 3:**
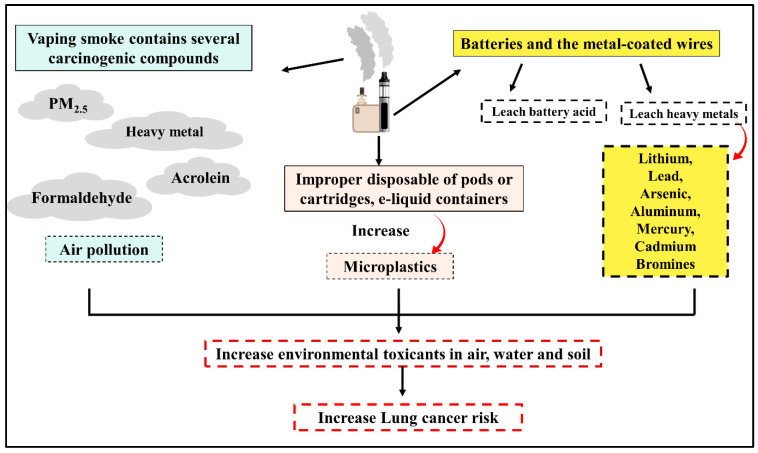
Relationship between using vape and rise of environmental toxicants exposure.

Co-exposure to various lung carcinogens could play a more synergistic or additive role in lung carcinogenesis than single carcinogen exposure [65,66]. Nowadays, the prevalence of vaping is on the rise accompanied by polluted air and contaminated environment. Additionally, the smoke from vaping contains several carcinogenic compounds that contribute to environmental contamination [67]. With the rapid increase in ECs users worldwide, secondhand exposure to ECs aerosols has become a serious public health concern [67]. It is proven that both smokers and second-hand exposure who live in contaminated environment are more prone to develop LC than others [67]. The incidence of LC is rising among nonsmokers or second-hand exposure which could be attributed to exposure to environmental carcinogenic compounds [10]. Importantly, more than 70 percent of inhaled E-cigarette aerosols are eventually exhaled, which may jeopardize active and negative smokers’ health [68]. The smoke from vaping contains several carcinogenic compounds that increase the risk of LC in both smoker and second-hand exposure. Vaping smoke and waste products share in environmental contamination and consequently increase the risk of LC. Exposures to specific environmental toxicants, either from vaping or other resources such as air pollution, heavy metals, and asbestos, have been reported to have a negative impact on pulmonary function and enhance lung carcinogenesis [10]. Exposure to PM_2.5_ is a well-established LC risk factor, and many studies have confirmed high concentrations of PM_2.5_ resulting from Ecs. In most cases, the reported indoor PM_2.5_ levels during Ecs use are above the WHO recommended threshold (25 μg/m^3^) [67,69]. Notably, a study detected 600 to 800 μg/m^3^ of PM_2.5_ concentrations in vape shops and vaping conventions [67,70]. The presence of carcinogenic aldehyde compounds such as acrolein, formaldehyde, and acetaldehyde in emitted smoke is detected in the indoor environment [69]. Acetaldehyde and formaldehyde are categorized by the IARC as possibly carcinogenic to humans (Group 2B) and carcinogenic to humans (Group 1), respectively [54]. Recently, electronic waste (e-waste) has received considerable attention due to its known risks to the environment and public health. Basic recycling processes release several toxicants, in particular, metals and microplastics, into the environment, impacting human health [71]. Electronic cigarette waste products such as (disposable vapes, pods or cartridges, e-liquid containers, and vape batteries) can leach contaminants into water, soil, and air [72]. Vape batteries and metal-coated wires can leach heavy metals (including lithium, lead, arsenic, aluminum, mercury, and bromines), battery acid, and nicotine into the local environment, affecting human health [67,73]. The impact of vaping smoke and waste products is exhibited in (Figure 3) Although the long-term effects of vaping on human health are not yet well established, the high levels of indoor air pollutants produced by E-cigarettes are raising alarms for public health.

## 5. Vaping and Lung Cancer

The term “vaping” refers to the usage of ECs or other devices to inhale a range of heated and aerosolized substances [74]. Vaping or ECs products can be used to deliver nicotine, flavorings, cannabis (CBD) and other chemicals [75]. Vaping devices were manufactured as nicotine replacement therapy to facilitate smoking cessation and decrease the negative health impact of traditional nicotine smoking [76]. The fundamental design of all vaping devices consists of three central components: a refillable or disposable liquid reservoir, a heating element, and a power source [15]. Classically, ECs convert a liquid solution comprising nicotine, VG, PG, and flavors into aerosols [77,78]. There are many various designs and styles available in the market ranging from disposable devices that resemble cigarettes to pod mods that are refillable and rechargeable using a USB cable [79,80]. Recently, modern devices such as JUUL have become more prestigious, rechargeable, stylized, controlled flavors, colorful, socially acceptable alternatives to conventional cigarettes, and equipped with attractive accessories. The rising trend of vaping among the public could be based on perceptions of the safety of flavorants and inhaling aerosol substances [81,82]. The absence of the production of carbon monoxide (CO) or other combustion-related toxic substances during vaping might increase its use. A vast improvement in the palatability of E-cigarette liquids (ELS) was observed; as nicotine-alone-based ELs have a bitter taste, there was a subsequent shift towards flavored ELs [15]. Furthermore, despite prior gains in lowering smoking rates and nicotine usage, the current data point to an alarming rise in vaping among younger people, especially adolescents [83]. From 2017 to 2018, high school students’ current usage of ECs increased from 11.7% to 20.8% and from 3.3% to 4.9% for middle school students. Current use of ECs is defined as having vaped within the previous 30 days [84]. Although the association between vaping and the development of LC is not well established, the carcinogenicity of EVPs such as nitrosamine compounds, humectants (PG and VG), flavoring compounds, CBD and vitamin E acetate has been attributed to several possible mechanisms.

### 5.1. Nitrosamine Compounds

Although vaping devices are marketed as safer than nicotine products, the correlation between ECs vaping and lung oncogenicity is still unknown [23]. It is documented that nitrosamine compounds attain adequate local concentrations within the distal bronchioles and alveoli, thus potentiating adduct formation and DNA damage [15]. The induced DNA methylation changes were supported by Lee et al.’s findings that ECs result from DNA adduct formation in murine bronchogenic tissues [85]. Tang et al. 2019, reported that mice exposed to ECs fumes for three months developed lung adenocarcinomas (9 of 40 mice, 22.5%); however, this tumor was particularly rare in mice exposed to filtered air or vehicle control. They suggested that ECs induces DNA damage in the lungs and inhibits DNA repair in lung tissues, implicate ECs as a lung carcinogen in mice [42]. However, another study found that stream air-vaporized nicotine is not lung carcinogenic in rats [86]. This discrepancy in findings could be explained by the fact that the aerosol size of ECs is smaller than the aerosols generated in traditional tobacco, and the small size of ECs aerosol allows nicotine to penetrate deeply into bronchioloalveolar cells [42]. Moreover, ECs induce mutagenic DNA adducts (cyclic 1,N2-γ-hydroxy-propano-deoxyguanosine [γ-OH-PdG] and O6-methyl-dG) in the mice lungs resulting in DNA damage [85]. It is suggested that tobacco smoke-generated ROS may result in lung epithelial cells’ DNA damage, prompting apoptosis and leading to the development of LC [87,88]. It has been that ECs induce similar respiratory epithelial toxicity and oxidative stress, which play a chief role in malignant transformation. This pathway appears to have multifactorial oncogenicity, with inflammation being directly associated with LC and with the adverse inflammasome/macrophage activation inducing an overall immunosuppressive “cold” environment, hostile to T-Cells—proven to be important in LC oncogenesis and malignant potential [89]. In addition, thermal breakdown of flavoring ELs creates carcinogenic organic aldehydes such as formaldehyde, acetaldehyde and acrolein [27,90]. It is recognized that formaldehyde, acrolein, and acetaldehyde are carcinogens [54,91,92].

Compared to conventional smoking, ECs create lower levels of carcinogens and toxic elements such as nicotine, PM, polycyclic aromatic hydrocarbons (PCA), formaldehyde nitrosamines, and heavy metals [15,25]. However, long-term exposure to low levels of the aforementioned elements poses serious health effects. Moreover, vaping devices can deliver substantial nicotine levels that could become addictive [25,93]. Several potential carcinogenic mechanisms could play a role in LC development, including increased DNA methylation, mutations, and binding to the nicotinic acetylcholine receptor that could induce tumorigenesis, survival, and invasion [94]. Furthermore, 80% of inhaled nicotine is metabolized into a nontoxic compound, cotinine which is excreted in urine [95]. At the same time, nearly 10% of inhaled nicotine undergoes endogenous conversion to nitrosamine compounds such as nitrosonornicotine and nitrosamine ketone [96,97]. These nitrosamine compounds are potent human carcinogens [54]. Tang et al. determined substantial nitrosamine ketone derivative levels 4-(methylnitrosoamino)-4-(3-pyidyl)-1-butanol in pulmonary tissues [98].

### 5.2. Propylene Glycol and Vegetable Glycerin 

Propylene glycol (PG) and vegetable glycerin (VG) are humectants commonly used in ELs with different ratios to produce aerosols that simulate traditional tobacco cigarette smoke [99]. The PG/VG ratio in the ELs is modified according to preferred plume (higher concentration of VG) or flavor (higher concentration of PG) [75]. The Food and Drug Administration (FDA) has approved the dermal or oral use of PG and VG in food, cosmetics and medications [100]. Although the FDA has categorized these humectants as generally recognized as safe (GRAS), the safety data of inhalation exposure to these elements and their thermal degradation products in ECs is limited [101]. The heated PG and VG in ECs are known to undergo thermal degradation, producing pulmonary irritants, free radicals and suspected carcinogenic carbonyl compounds (acetaldehyde, formaldehyde, and acrolein) [102]. It has been proposed that chronic PG/VG exposure damages epithelial barrier function by reducing lung cell volume and membrane fluidity, which could contribute to airway damage [103]. Many studies have observed that exposure to aerosols from PG and VG leads to an increase in pro-inflammatory and oxidative stress reactions [104,105,106]. A recent study found that PG is metabolized to methylglyoxal (MGO) in airway epithelia leading to altering mucociliary function via reduction of Ca^2+^ activated and voltage-dependent K^+^ (BK) channels [107]. It is suggested that MGO is a potent glycation agent in the human body resulting glycation of proteins, DNA and lipids and the gradual accumulation of advanced glycation end products (AGEs) in cells and tissues [108]. Recently, emerging evidence indicated that MGO plays a role in cancer development and progression [109]. Furthermore, Huynh et al. 2020 demonstrated the lung colonization-promoting effects of ECs on human breast cancer cells, indicating the risks of ECs on the lung metastasis of various cancers [110]. 

### 5.3. Flavoring Compounds

Vaping offers a diverse range of flavors, which is one of the main ECS attractions among youth and non-smokers [27]. The traditional mint flavor was recently replaced by a wide range of artificial contaminants and flavoring liquids with a high risk of pulmonary toxicities [25]. Several flavoring additives are aldehydes, and recent studies have examined the impact of toxic aldehyde emissions on human health during vaping [27]. There are growing concerns regarding the safety profile of ELs flavors compounds [111]. Importantly, ECs generate vapors using a heating element, which can lead to the decomposition of ELs ingredients. As coil temperatures rise, the liability of oxidation, pyrolysis, and thermal decomposition of organic aldehydes increases [100,101]. Thermal breakdown of flavoring ELs creates toxic organic aldehydes during vaping at high concentration levels that exceed occupational safety guidelines [90]. Numerous studies have exhibited the formation of toxic aldehydes, especially formaldehyde, in ECs vapors during vaping [27]. Gillman et al. discovered that the aldehyde emission levels in flavored ELs were 150–200% greater than those in unflavored e-liquids [112]. Many organic aldehyde derivatives are included (formaldehyde, acetaldehyde, acrolein, glyoxal, hexanaldehyde, and methanol) [27]. The data regarding the impact of ELs flavors mixture on inhalation toxicity and lung oncogenesis are insufficiently well established. Also, it is unclear whether the concentration limits are relevant to human exposure levels [111]. It is recognized that formaldehyde, acrolein, and acetaldehyde are carcinogens [54,91,92]. Several mechanisms have been suggested by which flavoring compounds in ECs could contribute to LC [15,27]. It is found that flavoring compounds in ELs induce intense toxic activity, an inflammatory response with macrophage activation and chemotaxis, vascular injury, dyslipidemia, and increased platelet reactivity) [113,114]. These inflammatory and toxic processes lead to the formation of reactive oxygen species (ROS) and encourage oxidative stress-induced lung tissue damage [115]. The ROS could be generated intracellularly (via mitochondrial oxidative phosphorylation) or from exogenous sources (E-cigarette aerosols or cigarette smoke) [116]. ROS plays a significant role in modulating the immune-inflammatory system and causes damage to DNA, [95] cellular membranes, lipids, and proteins [117,118]. Raised ROS levels could lead to the activation of polymorphonuclear neutrophils (PMNs), which generate further ROS in lung tissue [119]. A study by Zahedi et al. demonstrated increased epithelial-mesenchymal transition (EMT) in A549 CCL-185 lung cancer cells, with resultant increased invasive/metastatic potential, on exposure to various flavored ELs including menthol based [120]. Additionally, Nair et al. found that the pro-inflammatory effects of menthol were mediated directly via TRPM8, resulting in calcium influx in a BEAS-2B cell-line model [121]. Remarkably, altered intracellular calcium through TRPM8 has been previously shown to induce a neoplastic phenotype in LC [122]. Another suggested ECS adverse effect is direct suppression on pulmonary epithelial cilia movement leading to impairment of clearance of toxic particles and increase the risk of respiratory infections [123].

### 5.4. Cannabidiol (CBD) Vaping Products 

Not only nicotine is used in ECs, but other vaporized substances, especially cannabis derivatives, are widely used worldwide [124]. Cannabidiol (CBD) vaping products have become extensively accessible in the United state since their legalization in 2018. However, data are scarce on the relationship between LC risk and vaping cannabis. Other known risk factors for LC, such as chronic tobacco use and flavoring compounds, could confound with cannabis and play a chief role in LC carcinogenesis [125]. Even though most ELs contain nicotine, CBD and cannabinoid based ELs consumption has increased significantly, especially among the younger population [126]. The consumption rates jump by 16.6% in Canada, 13.8% in the US, and 9.0% in the UK [124,126]. It is unclear how vaping CBD negatively affects respiratory cell function. Cannabis ELs are prone to thermal decomposition and pyrolysis, yielding diverse potentially toxic organic compounds [127]. Cannabidiol vaping products oxidized into a reactive CBD quinone (CBDQ), which generates adducts with protein cysteine residues, altering protein function [112]. CBDQ was found to induce cytotoxicity, apoptosis in specific cells, liver toxicity, and inhibit topoisomerase II and angiogenesis. Thus, CBD can potentially have harmful adverse effects on lung cells [128]. It has been revealed that aerosolized CBD induces apoptosis, pro-inflammatory reactions, ROS generation, and enhanced cytotoxicity in bronchial epithelial cell lines [129,130]. The potential for cannabis oncogenicity could be attributed to toxic and pro-inflammatory effects on respiratory functions and can cause pulmonary irritation [15]. Notably, the meta-analysis of CBD smoking and LC risk by Zhang et al. failed to determine an increased risk of LC with cannabis use [131]. Another study by Thomas et al. explored that synthetic cannabinoid-based ECs result in significant and relatively unpredictable pyrolytic organic reactions [132]. 

Different oily substances were used in many ELs as thickening agents and diluents. Vitamin E acetate (VEA) was used as oil in ECs, mainly cannabinoid-based e-liquids [133]. Recent studies reveal that pyrolysis of cannabinoid and nicotine E-cigarette mixtures can produce hazardous toxicants whose synergistic actions potentially drive acute lung injury upon inhalation [133]. Emerging evidence indicates a remarkable rise in E-cigarette or vaping-associated lung injury (EVALI) among cannabinoid-based vaping users, characterized by acute lung injury or organizing pneumonia [134]. The US Centers for Disease Control and Prevention (CDC) documented 2807 EVALI cases in hospitals and sixty-eight deaths in the US [135]. Generally, VEA is a relatively safe biologically inert compound; however, several EVALI cases have recently been documented [136]. Notably, VEA has been determined in the bronchoalveolar lavage fluid of 29 patients diagnosed with EVALI. It is suggested that vitamin E acetate and other ECS compounds play a significant role in the pathogenesis of this injury [137]. Wu et al. proved that the thermal decomposition of VEA produces highly toxic and irritant ketene gas (via elimination of the aryl acetate group) along with several other toxic ROS with noticeable carcinogenic activity, including benzene and various alkenes [138]. The aforementioned thermal decomposition of VEA plays an important role in lung carcinogenesis. Wu and O’Shea’s study demonstrated that vaping VEA can lead to exposure to the toxic gas ketene [138]. In animal experiments, severe, acute lung damage was observed after 24 days of ketene exposure. The Acute Exposure Guideline Level (lethal) 10-min exposure value for ketene is 0.24 ppm [139]. Table 1 summarizes the common additives and pollutants in vaping fluid, their mechanisms of pulmonary damage, or related toxicities.

Increased use of E-cigarettes and ECs has raised numerous adverse health concerns involving the risks of heavy metals exposure via Els and vapors [145]. Current studies have confirmed that many heavy metals are present in both EC liquids and vapors at potentially harmful levels, which endangers both user and passive vaping [146]. Several metal levels have been detected in ECs, ELs, and human biological samples collected from vaping users [147]. The most commonly found metals were arsenic (As), copper (Cu), cadmium (Cd), chromium (Cr), lead (Pb), nickel (Ni), iron (Fe), and zinc (Zn) [148]. The source of these metals is commonly from the metal coils incorporated in the clearomizer of the ECs device or from e-liquids [149]. Mikheev et al. found that the previous metal presence in nanoparticle size was less than 2.5 µm [150]. Hence, the ultrafine size range is more dangerous to the lungs than larger ones due to their ready access to the alveolar region and rapid absorption systemically [151]. Heavy metal exposure in ECs is linked to significant health threats, such as neurotoxic and carcinogenic effects [152]. Chronic inhalation of lead nanoparticles is linked with respiratory and central nervous system pathological changes [153]. Co-exposure to several heavy metals in ECs caused oxidative stress as indicated by increases in the generation of ROS and the expression of ferritin light chain mRNA and heme oxygenase-1 mRNA and protein [141]. Heavy metals prompt apoptosis and evoke oxidative stress and DNA damage in lung cells [141].

The potential harmful consequences of E-cigarettes are also linked to respiratory system damage [154]. The published data regarding heavy metal exposure in ECs and the risk of LC is scarce. Because E-cigarette use is still relatively new, there may not have been enough opportunity to observe long-term impacts, including LC [154]. Significantly, Ni is classified as a respiratory carcinogen [54]., and the lung represents the most sensitive target of Ni toxicity [155]. Fowles et al. determined Cr and Ni in Els and aerosols and stated that prolonged exposure to Ni could substantially enhance the carcinogenesis process [154]. Hess et al. documented high levels of metal concentrations up to 400-fold in ECs, particularly Cd, Cr, Pb, and Ni in Els [156,157]. 

## 6. Environmental Toxicants and Lung Cancer

**Figure 4:**
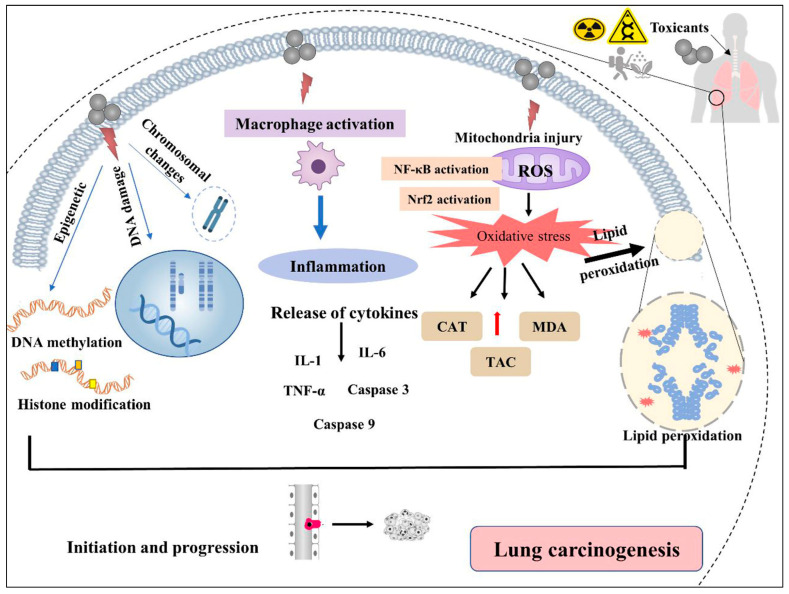
Mechanisms of environmental toxicants causing lung cancer. DNAdsb: double-strand DNA breaks, ROS: reactive oxygen species, NF-κB: nuclear factor kappa B, Nrf2: nuclear factor erythroid 2–related factor 2, IL-1/6 interleukin-1/6, TNF-α: tumor necrosis factor-alpha, Caspase: cysteine-dependent aspartate-directed proteases, CAT: Catalase, MDA: malondialdehyde, TAC: Total antioxidant capacity.

Human exposure to environmental toxic substances with different mechanisms of action is a growing concern [35]. Even though tobacco smoking is a potent lung carcinogen, a significant percentage of lung cancer mortality occurs in non-smokers. Other risk factors besides smoking can contribute to 15–25% of all LC of non-smokers; however, its epidemiology is poorly established [158,159]. It is recognized that various chemicals in environmental contexts have been proposed to affect health [160]. Exposure to known and probable respiratory carcinogens, such as metals and organic toxicants, is an essential enhancer of carcinogenesis [161]. Chronic pulmonary inflammation is a significant risk factor for LC tumorigenesis [162]. The association between environmental toxicants and LC in epidemiological evidence is poorly established [35]. However, numerous experimental investigations have demonstrated that several substances, such as heavy metals, ionizing radiation, pesticides, dust and fibers, household coal, arsenic, asbestos, and polycyclic aromatic hydrocarbons, can cause cellular and molecular changes that can facilitate the development of cancer [54]. The lifelong exposure to various toxicants, dosing, confounding variables, and human physiological diversity are essential issues in LC development [12,39,45]. Recently, the adverse effects of environmental toxicants on the lungs have been an area of intense investigation. Figure 4 summarizes the potential mechanisms by which the environmental toxicants can induce LC. However, it is worth noting that the environmental, dietary, and life habit factors could influence the molecular pathologies and pathogenic processes of LC [163,164,165,166]. For example, by reducing oxidative stress levels, DNA oxidative damage, and modulating epigenetics, some nutrients and phytochemicals could have antioxidant/anti-inflammatory properties that can affect or prevent LC pathogenesis [167,168]. It was evident that the use of dietary antioxidants and/or the nutritional supplements of vitamins and minerals in patients with cancer, including LC, has reduced the cancer growth/mutation rates and induced differentiation/de-differentiation [163]. A link between smoking and specific mutational signatures has been well known. Smoking could increase cancer risk by increasing the somatic mutation load, including base substitutions, indels, and copy number changes associated with the misreplication of DNA damage [169,170].

Additionally, the interplay between the mutational signature landscape of the tumor and other outdoor/indoor exposures in increasing the risk of lung cancer in never-smokers was confirmed in the “Sherlock-Lung study; 2018–ongoing” [171]. Collectively, these studies highlight the limitation of dependence on just one aspect in determining the risk, diagnosis, and therapeutic approach to patients with cancers, including lung cancer, in the era of precision medicine/personalized treatment [172,173].

### 6.1. Radiation

Exposure to radiation causes cancer; in particular, LC has been well-established in the literature. Previous research has demonstrated that all ionizing radiation groups are carcinogenic to humans [54]. The main radiation types linked to lung cancer include X-rays, γ-rays, and α-particles [174]. Regarding the source of radiation, medically related procedures account for 48% of the average individual’s radiation exposure in the US [175]. Current research revealed that exposure to diagnostic radiography was associated with an increased risk of LC.

#### 6.1.1. Radon

Radon is recognized as an essential natural environmental lung carcinogen (group 1), and the exposure occurs in occupational and outside the workplace [41]. Radon is a colorless, radioactive, odorless gas produced in the uranium-238 decay chain [176]. Radon is found outdoors, in soil, mines, dissolved radon in water, and in-built environments, such as offices, homes, and schools. Indoor radon is the most significant source of natural radiation to which humans are exposed (approximately 50%) [177]. Radon released from the soil into the atmosphere depends on the Uranium-238 content of the geological substrate on which these buildings are settled, soil parameters (porosity, density, and humidity), and weather conditions (wind, rain, and humidity, etc.) [178,179]. Importantly, radon is the second LC risk factor after smoking and may be responsible for 3–14% of LC cases, based on the WHO report [180]. Several previous publications documented a significant increase in LC mortality with cumulative exposure to radon and its decay products [181]. In Europe, nearly 21,000 deaths (2%) from cancer were attributed to radon [180,182]. Radon exposure in mining was linked to LC risk, as recognized by numerous investigations of non-smoking underground mine workers [183,184,185]. A study done by Darby et al. from 13 European case-controlled studies on 7148 cases confirmed a statistically linear increase of 16% (range, 5–31%) of LC risk per 100 Bq/m^3^ of indoor radon [182].

Radon emits α-ionizing particles, such as polonium-218 (^218^Po) and polonium-214 (^214^Po), associated with various genotoxic and cytotoxic effects. Although radon leads to DNA damage and genomic instability, the particular carcinogenesis mechanism in LC remains unidentified [186]. Alpha radiation releases energy, more than gamma and beta radiation. The released energy interacts with respiratory epithelium DNA differently, inducing DNA breaking, deletions, mutations, substitutions, and chromosomal changes [187,188]. The outcomes of genomic instability include cell cycle alteration, cytokine dysregulation, cell cycle regulation-related protein overexpression, apoptosis, and carcinogenesis [187]. Additionally, radon leads to oxidative stress and the release of ROS and hydroxyl radical attack [181,189]. Lim et al. detected genomic instability among high radon tumors in the forms of DNA damage and repair, such as *ATRX*, *ATR*, *RAD50*, *BARD1*, *TP53*, and *SMARCA4* [190]. In chronic radon exposure, Chen et al. found that mutant *KRAS* was overexpressed in bronchial epithelial cells, linked to oxidative damage and let-7 downregulation [191]. It is observed that high radon levels cause chromosomal arrangements and micronuclei in miners [187]. Moreover, epigenetic influences play a significant role in radon carcinogenesis, including DNA methylation, alteration of histones, and microRNA dysregulation [192]. On a molecular basis, the miRNA dysregulation associated with radon exposure involved the upregulation of microRNA-15 (miR-15), mirR-19, miR-16, and miR-23, as well as the downregulation of miR-369, miR-373, let-7, miR-124, miR-194, miR-652, and mirR-146 [192,193,194,195]. Consequently, these miRNA dysregulations result in altered DNA methylation, oxidative stress, cell cycle, inflammation, and malignant transformation in patients with LC exposed to radon [181]. Identification of the cellular and molecular basis of radon-induced LC is essential and would provide significant assistance in the reduction of radon-induced carcinogenesis. [187,192].

#### 6.1.2. Medical Radiation

Medical ionizing radiation exposure from diagnostic X-rays and radiation therapy γ-rays has been linked to different cancers. According to the IARC report, X-rays and γ-rays are classified as Group 1 lung carcinogens [54]. It is observed that stomach cancer is the first leading type of cancer, followed by LC, based on the Lifespan Study (LSS) of atomic bomb survivors in Nagasaki and Hiroshima, Japan [196]. Several studies found that risk estimates on second LC. Among patients who were treated between 1961 and 2007, 40% of them received radiotherapy; there was a significant association between radiotherapy and second LC with a RR of 1.23 (95% CI 1.07–1.43) [197]. A study explored the risk of radiation-induced LC in 10 years among women diagnosed with breast cancer who received radiation therapy. It is reported that the risk of LC overall was raised in females who underwent irradiation compared with those who were not irradiated, with a relative risk of 2.0 (95% confidence interval, 1.0–4.3) [198]. However, the evidence for ionizing radiation and LC relationship is inconclusive. Further studies that investigate the genetic radiation-molecular signature are recommended.

### 6.2. Air Pollution

The respiratory tract is a sensitive organ in contact with the external atmosphere and is liable to be exposed to air pollutants daily. Thus, a chronic inflammatory response and oxidative damage were provoked to deal with these foreign toxic particles, raising the risk of LC development [199,200]. Environmental exposures to harmful particles and gases, such as sulfur dioxide, ozone, and particulate matter (PM), have raised questions about the carcinogenesis of prolonged exposure to LC [201,202]. The WHO has concluded that there is an increased risk of lung cancer, with an estimated 250,000 worldwide deaths per year attributable to atmospheric pollution [203]. Although the association between air pollution and LC risk was studied in several epidemiological prospective research, this association was debatable, and other risk factors could be shared in LC [201]. A recent study discovered that high PM_2.5_ exposure, high genetic risk, and smoking were strongly linked to LC occurrence [48].

#### Airborne Particulate Matter (PM)

A complete understanding of how exposure to environmental air pollution provoked cancer development is lacking [204]. Air pollution exposure assessment has been challenging since several indoor and outdoor activities yield various air pollutants. Cooking and incense burning were considered significant indoor air pollutants linked to LC [205]. The “airborne particulate matter” (PM) terminology refers to a complex mixture of liquid and solid particles with varying compositions and sizes. PM is a significant component of air pollution and has been demonstrated to raise the risk of cancers and other diseases [206,207]. PM is categorized according to the diameter of the particle into coarse (2.5–10 μm, PM_10_), fine (0.1–2.5 μm, PM_2.5_), and ultrafine (≤0.1 μm, PM_1_). Different sources and human activities produce PM of various sizes, and PM has been extensively utilized in monitoring air pollution [208]. Long-term exposure to air pollution, especially particles with aerodynamic diameters ≤2.5 μm (PM_2.5_), has been a significant environmental risk factor for LC. Exposure to high levels of PM_2.5_ for a long time may cause inflammatory reactions and recurrent particle deposition, which can alter lung cell functions and increase the risk of LC [209]. According to IARC, PM was categorized as human carcinogenic (Group 1) in 2013 [54]. The adverse health effect of PM_2.5_ on LC has been well established, and a significant relationship between PM and incident LC has been observed in many studies [206,207,210,211]. In Rome, a 9-prospective study reported that the rise of each 10 μg/m^3^ PM_2.5_ level resulted in increases in LC Hazard ratio (HR) mortality of up to 6.2% [212], and these results were consistent with a US cohort research with an average follow-up of 8 years (HR = 1.33 for per 5 μg/m^3^) [211].

The association between LC of different histological subtypes and PM_2.5_ is yet underdeveloped, and future research focusing on this aspect is needed. One European Study of Cohorts for Air Pollution Effects (ESCAPE) determined that the HRs for LUAD and LUSC for the studied groups who did not change residence during the research period were 1.65 and 0.65 per 5 μg/m^3^ [213]. Similarly, a Canadian study observed that the HRs of LUAD and LUSC among females were 1.44 (95 % CI: 1.06, 1.97) and 1.28 (95 % CI: 0.74, 2.23) with a 10 μg/m^3^ increase in PM_2.5_ concentration [214]. In contrast, another study discovered no significant associations between PM_2.5_ and LC subtypes [215]. Numerous factors could play a role in PM_2.5_ carcinogenesis, such as the variable composition of PM_2.5_ in various locations, various exposure measurement techniques, different susceptibility to PM_2.5_, and durations of the study [48].

Many biological processes could be responsible for LC development, such as activating particular oncogenes mediated by microRNAs (miRs) leading to LC. It is suggested that PM_2.5_ encourages LC by acting on pre-existing oncogenic mutations in healthy lung cells [216]. Hill et al. reported a significant association between PM_2.5_ levels and the incidence of LC in 32,957 EGFR-driven LC cases. They investigated the sequences of tumor DNA samples from non-smokers living in polluted areas and reported that the sequences reveal few genetic point mutations, which can stimulate genes established to drive cancer growth [204]. Another study found that PM_2.5_ caused upregulation of the expression of three target oncogenes, namely serpin family B member 2, solute carrier family 30, member 1, and aldo-keto reductase family 1 member C1, which are significantly expressed in human LUSC [217].

### 6.3. Heavy Metals

Heavy metals are naturally existing metals having elemental densities larger than 5 gcm^−3^ and atomic numbers more than 20 [218]. Global industrialization, rapid urbanization, and mismanagement of wastes have imported heavy metals into the living environment [219]. Importantly, improper, uncontrolled disposal of industrial effluents leads to the presence of these heavy metals in water, land, and air [220]. Heavy metals are significant environmental toxicants globally, and their toxicity is a major public health concern [221]. Health adverse effects from metals in food, water, and air have been reported [1,2,3,4,5,6,7,8,9]. Heavy metals include industrial or electronic wastes, gasoline and diesel engine exhausts, pesticides, paints, incinerators, and agricultural products; they can be easily absorbed via ingestion, inhalation, or dermatological routes [6]. A large quantity of metal is used in electronic products, such as nickel (Ni), cadmium (Cd), chromium (Cr), and arsenic (As), which increases the risk of LC [222]. As a result, there is a possibility that the high metal concentrations released from e-waste could deteriorate health [223]. Although many essential metals, such as iron, magnesium, copper, and zinc, are required in recommended concentrations for human body biological processes, higher concentrations may be harmful [224]. Contrarily, other heavy metals, namely As, Cd, Cr, and Ni, have no detectable effect on biological activities and have been associated with the increased risk of LC [225,226,227].

#### 6.3.1. Cadmium (Cd)

Cadmium is a category I pulmonary carcinogen that is a ubiquitous environmental pollutant with a highly toxic impact on human beings. Cadmium targets the liver, testes, lungs, and kidneys following acute and chronic intoxication and also stimulates tumorigenesis in prolonged exposures [228]. Exposure to Cd occurs through the inhalation of tobacco smoke or polluted air and the ingestion of contaminated water and food [229]. It is known that smoking cigarettes is the most common source of Cd exposure, and Mona et al. observed a significant increase in Cd concentration in smokers’ urine and serum compared to non-smokers [230]. Environmental and occupational Cd exposure has been associated with breast, nasopharynx, lung, prostate, urinary bladder, and pancreas cancers [231]. Lee et al. observed that participants who lived in highly polluted zones suffered from an increased prevalence of LC and a nearly 1.25-fold rise in the incidence of the risk of LC for each 1 μg/g-creatinine increase in the urine Cd level. Also, they found that patients with LC had significantly higher urinary Cd levels with a worse prognosis [227]. Another meta-analytical study stated that an estimated 68 percent increase (*p* < 0.0001) in the relative risk of LC was observed with a doubling of urinary Cd [232]. The association between Cd levels and Lc histological types is unclear; however, Demir et al. detected high Cd levels in patients with advanced stages of squamous and large cell LC compared with remaining LC types [233]. Another study reported that patients with squamous cell LC had greater urinary Cd concentration [227].

It has been known that Cd increases oxidative stress in ROS generation and provokes inflammatory cytokine production, contributing to respiratory tract irritation and pulmonary edema. Furthermore, Cd-induced apoptosis could be induced by Ca^2+^ accumulation, ROS production, Bcl-2 reduction, and apoptotic genes (e.g., *Bcl-2*, *P53*, *Bax*, *Caspase 3*, and *Caspase 9*) dysregulation [228,234]. In addition, Cd altered mitochondria functions in various pathologies, including oxidative stress and producing ROS, triggering apoptosis, altering gene expression, mutating mtDNA, insufficiency ATP release, and lipid peroxidation [235]. In animal studies, Cd leads to genotoxicity via DNA strand breaks, mutations, chromosomal damage, impaired DNA repair, and cell transformation [236].

#### 6.3.2. Arsenic (As)

Arsenic is a highly toxic category I carcinogen metal responsible for different toxicity mechanisms and harmful effects on human organs, particularly the lungs [237]. Arsenic exposure can occur through ingestion and inhalation, respectively, in the form of soluble arsenite and particulate arsenic trioxide. Soluble arsenite has been proven to induce LC via both routes [238]. Long-term inhalation of inorganic As can cause chronic arsenic poisoning, which can cause hyperkeratosis, skin lesions, and, in some cases, bladder and lung cancer. Long-term inhalation of As can cause chronic As poisoning, which can cause hyperkeratosis, skin lesions, and bladder and lung cancer [239]. Smoking cigarettes and occupational exposure contributed to the elevated levels of inorganic As exposure [240]. The synergic interaction between smoking and As exposure could result in a significantly higher risk of LC [241].

The American Conference of Governmental Industrial Hygienists (ACGIH) demarcated the Threshold Limit Values (TLV-TWA) as 0.01 mg/m^3^ for As and inorganic compounds and 0.005 ppm for arsine. At the same time, the National Institute for Occupational Safety and Health (NIOSH) determined that the recommended exposure limit (REL) for arsine is 0.002 mg As/m^3^ [242]. The metabolism of As most likely contributes significantly to its carcinogenicity [243]. Arsenic is mainly metabolized by methylation into trivalent and pentavalent forms, considered toxic reactive metabolites. Reduced methylation capacity could result in a rise in LC risk, particularly in the presence of high levels of As [244].

The adverse health effect of inorganic As exposure could be delayed for up to four decades [245], and various studies suggest that exposure to As during gestation and childhood could result in LC in adulthood [246,247]. Several possible pathologies are linked to As-induced carcinogenesis, including oxidative epigenetic modification, DNA damage, genetic instability, and immunomodulation [237]. It is commonly accepted that oxidative stress can lead to the development of oxidative DNA damage, which is a factor in carcinogenesis [248]. Recently, Islam et al. found that miR-218-5p and EGFR were significantly downregulated and upregulated in arsenic-induced transformed (As-T) cells, respectively. It is suggested that tumor-suppressive miR-218-5p suppresses cancer proliferation, migration, and angiogenesis. In addition, miR-218-5p specifically targeted EGFR by attaching to its 3′-untranslated region (UTR) [249]. Overexpression or mutation of *EGFR* has a critical role in carcinogenesis in NSCLC [250]. Hence, EGFR is a known cancer biomarker frequently expressed in LC, while miR-218-5p possesses antitumor activity through EGFR. The miR-218-5p/EGFR signaling pathway plays a pivotal role in the increased risk of LC [251]. Recent research suggests impaired host immunity, specifically T cell anti-tumor immunity, may be crucial for cancer development [237,252,253]. A recent study reported that prolonged exposure to As in drinking water up-regulated programmed death-1 (PD-1)/programmed death-ligand 1 (PD-L1) (PD-1/PD-L1), increased regulatory T cells (Tregs), decreased the CD8/Treg ratio, and these changes in mice’s lungs enhanced the formation of LC. CD8 acts as an anti-tumor immunosuppressive, while PD-1 and its ligand, PD-L1, are known as T cell inhibitory receptors [252]. It is proven that inhalation of particulate arsenic trioxide produces significant DNA damage in lung epithelial cells via strand breaks, oxidative damage, and superoxide formation [254].

### 6.4. Asbestos

Asbestos is a significant environmental carcinogen connected to lung cancer and has been identified by the IARC as one of the lung carcinogens group 1 [54]. According to WHO estimates, 125 million individuals are globally exposed to asbestos, which can lead to LC, laryngeal cancer, and mesothelioma development [255]. In the last few years, there has been a growing interest in asbestos-induced lung cancer. Asbestos fibers are naturally occurring silicate mineral fibers that have been heavily used in manufacturing due to their extraordinary properties. In buildings and construction, asbestos has been used as a thermal insulator in ceilings, flooring, shingles roofing, fire-retardant coatings, and water pipes and as an additive for asphalt concrete to improve the road surface’s stability [54]. Globally, asbestos-related lung disease is a severe public health issue, and inhaling asbestos fibers increases the risk of developing LC and malignant mesothelioma [256,257]. High levels of asbestos are the primary cause of between 5% and 7% of all LC-reported cases worldwide, chiefly due to occupational exposure [258]. Asbestos is responsible for around half of the occupational cancer deaths among workers in the asbestos sector [259]. Not only does occupational asbestos exposure lead to LC but also household exposure is responsible for thousands of deaths yearly due to the wide use of asbestos and asbestos-containing fibers in the home [260,261].

Additionally, it has been demonstrated that asbestos fibers and cigarette smoke exposure considerably raise the risk of LC. The danger increases when a person smokes more [262]. Interestingly, asbestos fibers trapped tobacco particulates, justifying the synergistic effect of asbestos with tobacco use on LC, with a cohort study reporting a 14.4-fold increased risk [263].

Several factors play an essential role in asbestosis and the risk of lung cancer, such as pulmonary functions, type and concentration of fiber, duration of exposure, genetic sustainability, and individual immunity. Long-term asbestos exposure can accumulate fibers in the lung tissues, causing chronic bronchitis, fibrosis, and pneumoconiosis (silicatoses) [228]. Additionally, various molecular abnormalities that may develop from asbestos exposure contribute to the worsening of the diagnosis and prognosis of LC [264]. Importantly, asbestos generates a considerable amount of ROS, which starts the genotoxicity cascade [24,25]. Although the precise methods by which asbestos damages DNA and induces apoptosis are not well understood, some of the pathways that have been suggested include the alteration in mitochondrial function, generation of ROS, reactive nitrogen species (RNS), and activation of the death receptor pathway [265].

Furthermore, oxidative stress may encourage cell death, gene mutations, and chromosomal abnormalities, ending in cell transformation. The sources of ROS production are attributed to the immune system’s response, fiber surface reactivity, and mitochondrial dysfunction [266]. Inflammation is another primary source of ROS synthesis. Asbestos triggers the production of ROS by alveolar macrophages and neutrophils during phagocytosis, a process that results in the secretion of proteases, chemokines, and cytokines, which mediate the inflammatory response [257].

It is well-recognized that microRNAs regulate a wide range of biological functions, including cell division, proliferation, and differentiation. In addition, changes in the expression profile of microRNAs are an essential epigenetic mechanism that may be involved in the pathogenesis of asbestos-induced LC [261]. Santarelli et al. stated that four serum miRNAs, namely miR-205, miR-520g, miR-126, and miR-222, were discovered to be associated with asbestos-related malignant diseases, and the authors proposed that these miRNAs are possibly contributing to LC linked to asbestos. Their expression reveals potential pathogenic mechanisms for asbestos-induced carcinogenesis [267]. 

A comparison of data from asbestos-exposed and MM subjects found that the most promising candidates for a multimarker signature were circulating miR-126-3p, miR-103a-3p, and miR-625-3p combined with mesothelin. The most consistently described tissue miRNAs, miR-16-5p, miR-126-3p, miR-143-3p, miR-145-5p, miR-192-5p, miR-193a-3p, miR-200b-3p, miR-203a-3p, and miR-652-3p, were also found to provide a diagnostic signature and should be further investigated as possible therapeutic targets. Similarly, Micolucci et al. found that asbestos increased some circulating miR-103a-3p, miR-126-3p, and miR-625-3p in combination with mesothelin, which were the most promising biological cancer markers; they also observed that asbestos significantly dysregulated the expression of miR-16-5p, miRNAs, miR-143-3p, miR-652-3p, miR-203a-3p, miR-126-3p, miR-192-5p, miR-145-5p, miR-193a-3p, and miR-200b-3p in LC [268]. Conclusively, these microRNAs increased in response to genotoxic stress, and their expressions aid in the diagnosis, follow-up, prognosis, and targeted therapy.

### 6.5. Pesticides

Pesticides, including insecticides, herbicides, fumigants, fungicides, and rodenticides, are crucial chemicals that are frequently employed in agriculture and other sectors. Organophosphate pesticides (OPPs) such as parathion, malathion, chlorpyrifos (CP), monocrotophos (MCP), and others have been extensively used in public health, agricultural, industrial, veterinary, and household contexts [269]. Molecular and epidemiological studies have demonstrated that OPPs are linked with increased cancer risk; however, the underlying mechanisms are not yet well developed [270]. Although many nations ban their use, OPPs are still used worldwide due to their cheapness, availability, and high efficacy. The improper use of huge uncontrolled applications has been identified in the environment as a pollutant [271]. Most OPPs feature a phosphorothioate group (P = S), which is safer than those with the P = O linkage because it has less reactivity with biomolecules and is hydrolytically stable [272]. Although many studies have identified the link between exposure to OPPs and the risk of LC, the precise carcinogenesis of these compounds is not yet well known [270,273,274,275]. A prospective study that was a component of the Agricultural Health Study (AHS) offered more proof in favor of a connection between lung cancer risk and pesticide use [274]. Jones et al. observed an increased LC incidence among men dealing with pesticides with high exposure over their lifetime days (diazinon: rate ratio, 1.60; 95%) [276]. Pesatori et al. found positive relationships between pesticide exposure and LC in a small, case-control study of structural pesticide applicators in Florida (odds ratio, 2.4; 95% CI, 1.0 to 5.9) [277]. Similarly, a French farmer cohort research found links between exposure to pesticides and the incidence of small-cell lung cancer (HR, 2.38; 95% CI, 1.07 to 5.28) [278].

Environmental and in vivo evidence indicates that OPPs can change from thionates to oxons, and thus, they become more toxic and reactive following exposure after inhalation, ingestion, and absorption from the skin [279]. The OPP action is based on irreversible acetylcholinesterase (AChE) inhibition in muscle and nerve tissues, causing the accumulation of acetylcholine in postsynaptic muscarinic and nicotinic choline receptors [280]. Several mechanisms are proposed in the literature to explore the pathogenesis of OPPs-induced LC. It is proven that OPPs play an essential role in oxidative stress, the generation of ROS, and reduced antioxidant enzymes, which consequently lead to oxidative DNA damage, altered DNA repair, and stimulated apoptosis [273]. Oxidative stress and oxidative DNA damage facilitate apoptotic signaling cascades in the mitochondria, further contributing to the release and activation of mitochondrial proteins such as apoptosis-inducing factor (AIF) and caspase-3 [281,282]. 

## 7. Conclusions

This article reviews how environmental risk factors and vaping may raise the risk of lung cancer. Epidemiological and experimental studies have shown that numerous chemical groups found in both vaping and environmental may adversely disrupt pulmonary functions and initiate carcinogenesis in an additive or synergistic manner. There is strong evidence that tobacco and flavoring compounds contribute to LC. Exposure to many toxicants found in vaping products and the environment, such as certain metals, suggests that these substances may also pose a risk for tumorigenesis. Risk factors for LC include hazardous geographic conditions such as places exposed to air pollution and natural radiation. Prenatal exposure to environmental toxicants during pregnancy or childhood may play a role in determining the development and severity of lung cancer in adulthood. Oxidative stress, the generation of ROS, oxidative DNA damage, apoptosis, and dysregulation of inflammatory proteins are the most common mechanisms that enhance lung carcinogenesis. The gene expression of several microRNAs plays a significant role in diagnosis, follow-up, and targeted therapy. However, more investigation is necessary to comprehend the mechanisms that result in lung cancer entirely and, ultimately, to identify the exact environmental toxicants that raise the risk of disease in individuals. The increased use of ECs and cannabis vaping, especially among young individuals, females, and nonsmokers, is a significant public health concern. Environmental effects from improper e-waste disposal and recycling are increasing worldwide. Electronic waste handling and disposal exposes people to highly toxic compounds, such as heavy metals. Air pollution, mainly PM_2.5_ from global industrialization and vehicle exhaust emissions, has been linked to LC. Consequently, reducing PM_2.5_ concentrations in the general population could be an effective preventative measure against LC. Even though occupational LC may have decreased recently, preventive measures are still required to lessen the exposures’ carcinogenic consequences. 

Future epidemiologic research and review on the relationship between lung cancer risk and prolonged use of E-cigarettes and exposure to other environmental factors with their molecular pathology and clinical consequences is highly recommended. These “molecular pathologic epidemiology” studies will enrich our understanding of “how a specific exposure could impact the process of carcinogenesis, somatic molecular alterations, and tumor biomarkers”, as proposed by others [283,284].

## Figures and Tables

**Table 1:** The common additives and pollutants in vaping liquid, their mechanisms of pulmonary damage, or related toxicities.

Compounds	Experimental Model of the Study	Dose Range	Protein/Gene Level Regulation, Signaling Pathway Activation	Final Outcome	Reference
Nicotine derivatives (Nitrosamine compounds)	E-cigarette smoke-exposed Mice. Cultured human bronchial epithelial and urothelial cells. In vitro DNA damage-dependent repair synthesis assay.	Mice: (10 mg/mL, 3 h/d, 5 d/wk) for 12 wk	↓ DNA-repair activity and repair proteins XPC/OGG1/2 in the lung. ↑ mutational susceptibility and tumorigenic transformation of cultured human bronchial epithelial cells	DNA damage, DNA methylation changes, and adduct formation. Increased the lung cancer risk.	[85]
E-cigarette JUUL pod flavors “(Fruit Medley, Virginia Tobacco, Cool Mint, Crème Brulee, Cool Cucumber, Mango, and Classic Menthol)” and similar pod flavors (Just Mango-Strawberry Coconut and Caffé Latte)”.	Lung epithelial cells (16-HBE, BEAS-2B) and monocytes (U937) exposed to various pod aerosols	In-vitro aerosol exposure system: “66 puffs during 22 min with a three-second puff duration at 1.6 L/min flow rate and an inter-puff interval of approximately 17 s.”	↑ acellular ROS ↑ mitochondrial superoxide production in bronchial epithelial cells (16-HBE). ↑ inflammatory mediators, such as IL-8/PGE2 in lung epithelial cells (16-HBE, BEAS-2B) and monocytes (U937)	JUUL pod flavors, “Crème Brulee and Cool Cucumber”, caused epithelial barrier dysfunction in 16-HBE cells. DNA damage upon exposure in monocytes Increase oxidative stress, inflammation, epithelial barrier dysfunction, and DNA damage in lung cells.	[88]
Vaping E-cigarette components	Total RNA from nasal scrape biopsies was analyzed using the nCounter Human Immunology v2 Expression panel.	Active E-cigarette users/vapers who have been using E-cigarettes regularly for at least six months	The top five genes with changed expression in E-cigarette users were Zinc Finger and BTB Domain Containing 16 (ZBTB16), EGR1, Polymeric Immunoglobulin Receptor (PIGR), Prostaglandin-Endoperoxide Synthase 2 (PTGS2), and FK506 Binding Protein 5 (FKBP5).	↓ Expression of immune-related Genes at the level of nasal mucosa.	[118]
Menthol or tobacco-flavored EC liquids or aerosols	Human adenocarcinoma alveolar basal epithelial cells (A549). Live cell imaging, Epithelial-to mesenchymal transition (EMT) biomarker analysis, and machine learning/image processing algorithms.	exposure to EC liquids and aerosols from a popular product for 3-8 days.	EMT is accompanied by the acquisition of a fibroblast-like morphology, loss of cell-to-cell junctions, internalization of E-cadherin, and increased motility. Upregulation of EMT markers. Plasma membrane to nuclear translocation of â-catenin	An EMT of lung cancer cells during exposure to EC products	[120]
Aerosolization of commercial Cannabidiol (CBD) vaping products	Click chemistry and a novel in vitro vaping product exposure system (VaPES).	A human bronchial epithelial cell line (16HBEs) was exposed to synthetic a-CBD or a-CBDQ (Quinone) for 4 h at a range of concent. (2−35 μM).	A reactive CBD quinone (CBDQ) forms adducts with cysteine residues in human bronchial epithelial cell proteins, including “Keap1” and activates “KEAP1-Nrf2” stress response pathway genes.	Vaping CBD alters protein function and induces cellular stress pathways in the lung.	[128]
49 commercially available e-liquid flavors	Free radicals generated from the flavors were captured/analyzed by electron paramagnetic resonance (EPR). The flavorant composition of each e-liquid was analyzed by gas chromatography mass spectroscopy (GCMS).	The flow meter of the E-cigarette setup was connected to the house vacuum and adjusted to a flow rate of 500 mL/min.	Nearly half of the flavors modulated free radical generation. Ethyl vanillin inhibited the radical formation in a concentration dependent manner. Free radical production was closely linked with the capacity to oxidize biologically relevant lipids.	Flavoring agents could enhance/inhibit the free radicals’ production in flavored E-cigarette aerosols. Some flavorants ↑ lipid peroxidation products. Some flavorants ↑ formation of 8-isoprostane (the oxidation products of arachidonic acid).	[140]
Ethyl maltol (EM; sweet flavor)	The Calu-6 and A549 lung epithelial cell lines co-exposed to EM and copper (Cu)	EM at 3 mM concentration as it was not toxic.	Cell viability DNA damage response Reactive oxygen species generation Ferritin light chain and heme oxygenase 1 mRNA upregulation	Co-exposure to EM and Cu at concentrations not toxic for either chemical individually induces oxidative stress, apoptosis, and DNA damage in lung epithelial cells.	[141]
Aldehydes	Different organs of mice exposed to mainstream tobacco smoke (MTS). Immortalized human bronchial epithelial cells BEAS-2B and urothelial cells UROtsa. Buccal cells and sputum. Lung tissues of tobacco smokers obtained from the marginal non-cancerous lung tissue samples of cancer patients.	Exposure of mice to MTS (75 mg/m^3^) for 6 h/d, 5 d/wk for 12 wk.	DNA Damage markers DNA Repair proteins determination. PdG adducts formation in human bronchial epithelial and urothelial cells	DNA damage DNA adducts formation and impairment of DNA repair proteins and activity.	[142]
Vitamin E acetate (VEA)	Human bronchial epithelial cells (BEAS-2B).	VEA vaping emissions were generated using a 0.46 L min-1 critical orifice to restrict the flow rate. Emissions were vaped into a glass cold trap submerged in dry ice; condensed emissions were dissolved in acetonitrile (ACN) for chemical analysis and cell culture media for cell exposure analysis. HMOX-1 and NQO1 gene expression analysis after 0, 3, 6, 12, and 24 h exposure to VEA vaping emissions.	Exposure to vaping emissions resulted in significant upregulation of NQO1 and HMOX-1 genes in BEAS-2B cells.	Oxidative damage Acute lung injury Synergistic interactions between thermal decomposition products of VEA could be evident, highlighting “the multifaceted nature of vaping toxicity”.	[143]
CBD/counterfeit vape cartridges and their constituents vitamin E acetate (VEA) and medium-chain triglycerides (MCT).	Bronchial epithelial cells (BEAS-2B). In Vivo Mouse Exposures with mouse arterial oxygen saturation and bronchoalveolar lavage (BALF) collection.	For in vitro exposures, cell culture plates were exposed to two 70 mL puffs of the aerosol under air-liquid interface conditions for 10 min. For in vivo exposures, wild-type mice with C57BL/6 background were exposed to 1 h MCT, VEA, and cartridge aerosols with 70 mL puffs, two puffs/min using the Scireq inExpose system	↑ IL-6, eotaxin, and G-CSF in BALF. ↑ Eicosanoid inflammatory mediators and leukotrienes in mouse BALF. ↑ hydroxyeicosatetraenoic acid (HETEs) and various eicosanoid levels in plasma from E-cig users. ↑ Surfactant-associated protein-A (SP-A) in lung homogenates from male mice exposed to VEA.	Acute exposure to specific vape cartridges induces in vitro cytotoxicity, barrier dysfunction, and inflammation. In vivo, mouse exposure induces acute inflammation with elevated proinflammatory markers. Prolonged exposure may cause significant lung damage, which is involved in the pathogenesis of E-cigarette or vaping products-associated lung injury (EVALI).	[144]

## Data Availability

Not applicable.
